# New and Old Genes Associated with Primary and Established Responses to Cisplatin and Topotecan Treatment in Ovarian Cancer Cell Lines

**DOI:** 10.3390/molecules22101717

**Published:** 2017-10-13

**Authors:** Monika Świerczewska, Andrzej Klejewski, Karolina Wojtowicz, Maciej Brązert, Dariusz Iżycki, Michał Nowicki, Maciej Zabel, Radosław Januchowski

**Affiliations:** 1 Department of Histology and Embryology, Poznan University of Medical Sciences, Święcickiego 6 St., 61-781 Poznań, Poland; m_swierczewska@wp.pl (M.Ś.); kwojtowicz@ump.edu.pl (K.W.); mnowicki@ump.edu.pl (M.N.); mazab@ump.edu.pl (M.Z.); rjanuchowski@ump.edu.pl (R.J.); 2 Department of Nursing, Poznan University of Medical Sciences, Smoluchowskiego 11 St., 60-179 Poznan, Poland; 3 Department of Obstetrics and Womens Diseases, Poznan University of Medical Sciences, Smoluchowskiego 11 St., 60-179 Poznan, Poland; 4 Division of Infertility and Reproductive Endocrinology, Department of Gynecology, Obstetrics and Gynecological Oncology, Poznan University of Medical Sciences, Polna 33 St., 60-535 Poznań, Poland; maciejbrazert@ump.edu.pl; 5 Department of Cancer Immunology, Poznan University of Medical Sciences, Poland, Garbary 15 St., 61-866 Poznań, Poland; dmizy@ump.edu.pl; 6 Division of Histology and Embryology, Wrocław Medical University, Chałubińskiego 6a, 50-368 Wrocław, Poland

**Keywords:** ovarian cancer, cisplatin and topotecan resistance, new genes

## Abstract

Low efficiency of chemotherapy in ovarian cancer results from the development of drug resistance. Cisplatin (CIS) and topotecan (TOP) are drugs used in chemotherapy of this cancer. We analyzed the development of CIS and TOP resistance in ovarian cancer cell lines. Incubation of drug sensitive cell lines (W1 and A2780) with cytostatic drugs was used to determine the primary response to CIS and TOP. Quantitative polymerase chain reaction (Q-PCR) was performed to measure the expression levels of the genes. We observed decreased expression of the *MCTP1* gene in all resistant cell lines. We observed overexpression of the *S100A3* and *HERC5* genes in TOP-resistant cell lines. Increased expression of the *S100A3* gene was also observed in CIS-resistant A2780 sublines. Overexpression of the *C4orf18* gene was observed in CIS- and TOP-resistant A2780 sublines. A short time of exposure to CIS led to increased expression of the *ABCC2* gene in the W1 and A2780 cell lines and increased expression of the *C4orf18* gene in the A2780 cell line. A short time of exposure to TOP led to increased expression of the *S100A3* and *HERC5* genes in both sensitive cell lines, increased expression of the *C4orf18* gene in the A2780 cell line and downregulation of the *MCTP1* gene in the W1 cell line. Our results suggest that changes in expression of the *MCTP1*, *S100A3* and *C4orf18* genes may be related to both CIS and TOP resistance. Increased expression of the *HERC5* gene seems to be important only in TOP resistance.

## 1. Introduction

One of the main reasons for the low effectiveness of chemotherapy in neoplastic disease is the development of drug resistance. A very good model to study this phenomenon is epithelial ovarian cancer (EOC), the most lethal gynecological malignancy [1]. Most patients respond well to chemotherapy. However, during contact with cytotoxic agents, patients of ovarian cancer develop resistance to these drugs [1,2]. In the case of EOC, the first line of chemotherapy is always composed of platinum and taxane [3]. Unfortunately, approximately 80% of patients with advanced ovarian cancer, from whom good response was obtained after the first-line of treatment, will have a recurrence and will require a continuation of the treatment. Based on their response to cisplatin (CIS), patients can be divided into the following groups: sensitive to platinum—recurrence after 12 months or more (54.9%); partially sensitive to platinum—recurrence within 6–12 months after completion of treatment (22.7%); not sensitive to platinum—recurrence within six months after treatment (17.2%); and resistant to platinum—lack of remission or progression during treatment (5.3%). The sensitive group can be further divided into patients who were probably cured (120 months without recurrence −17.7%), sensitive with progression within 60–120 months after treatment (3.7%) and sensitive with progression within 12–60 months after treatment (33.5%). Depending on the recurrence subgroup, one can administer taxane derivatives, CIS, topotecan (TOP), liposomal doxorubicin, or gemcitabine [4,5,6]. For most drugs, the response to second-line chemotherapy amounts to 15–35%.

CIS is the most frequently used antitumor agent and is used in lung, testis, and ovarian cancer chemotherapies, among others. CIS inhibits DNA replication and RNA transcription by interacting with the nitrogen atoms of the DNA, preferentially with the N-7 atom of deoxyguanylic acid, which results in intrastrand and interstrand DNA cross-linking [7]. Cancer cells develop different forms of resistance to CIS, and the most important of these are the repair of damaged DNA via DNA repair systems [8], decreased drug uptake [7], increased reflux by drug transporters of the ABC family such as ABCC2 (MRP2) [9], and increased drug inactivation by sulfhydryl-containing molecules such as metallothioneins [10] and glutathione [11].

Topotecan, a semisynthetic derivative of camptothecin [12], is an inhibitor of DNA topoisomerase I, a nuclear enzyme that regulates overwinding or underwinding of the DNA helix [13,14]. TOP acts by stabilizing the enzyme-DNA complex, resulting in the inhibition of DNA replication and transcription, leading to cancer cell death [15]. The most significant mechanism of resistance to this drug is active transport from cancer cells. The most important protein playing a role in this phenomenon is BCRP (breast cancer resistant protein) encoded by the *ABCG2* gene [16,17,18,19]. Previously, we reported that the expression of the *ABCB1* (*MDR1*) gene, encoding the P-gp protein, may also be related to TOP resistance [18,19,20]. Another important mechanism of TOP resistance involves mutations in DNA topoisomerases or decreased expression of these enzymes, making them less sensitive to drugs [21]. Our recent observation indicates that both CIS and TOP resistance may also be associated with increased expression of different collagen genes [22,23,24].

However, new genes that may be related to drug resistance are still being discovered. Our microarray results indicated that the *MCTP1*, *S100A3*, *C4orf18* and *HERC5* genes may also be involved in drug resistance in ovarian cancer [25]. MCTP1 (multiple transmembrane protein 1) contains three C2-domains with high Ca^2+^ activity and contains two transmembrane regions [26]. The C2 domain is Ca^2+^ binding motif present in proteins involved in membrane trafficking/exchange processes and is critical for vesicle formation, receptor trafficking, cell migration, and neurotransmitter release [27]. Differences in the expression of MCTP1 were observed in colorectal cancer specimens [28]. The *S100A3* protein is a member of the S100 protein family, which is comprised of 22 members. S100 proteins are localized in the cytoplasm and nucleus in different cell types and are involved in cell-cycle progression and differentiation [29]. These proteins contain two EF-hand calcium-binding motifs connected by 10–12 residues, forming a critical hinge-like region (loop 2) involved in interactions with the target [30,31]. Expression of *S100A3* has been reported in many cancers. In gastric, colorectal, and hepatocellular cancers, the expression of *S100A3* is upregulated [32,33,34]. Furthermore, *S100A3* expression is correlated with tumor differentiation and TNM in gastric cancer [32]. C4orf18—FAM198B is a poorly described protein. According to different databases, its expression has been detected in nerves and in the epithelium during development. To our knowledge, its expression has not been described in the PubMed database thus far. HERC5 (HECT Domain and RCC1-Like Domain-Containing Protein 5, HECT-type E3 protein ligase) is an interferon-induced E3 protein ligase that mediates the ISGylation of protein targets [35,36]. This enzyme transfers ISG15 protein from an E2-conjugating enzyme such as UbcH8 to a specific protein substrate [35,36]. ISGylation of target proteins probably leads to degradation by 20S proteasomes [37]. It has been reported that HERC5-dependent p53 ISGylation plays a role in p53 inactivation during oncogene-mediated transformation [38]. Expression of HERC5 and ISGylation affects the proliferation of cells in prostate cancer, indicating their role in malignant transformation [39].

Drug resistant studies, in most cases, are conducted on pairs of drug-sensitive and resistant cell lines, where cells have been exposed to cytotoxic agents for a few months or more. Knowledge about the response to these agents during the first days of treatment is scarce. The goals of our study were as follows: (1) to compare the expression levels of new genes involved in CIS and TOP resistance in drug-sensitive and drug-resistant ovarian cancer cell lines; and (2) to establish the expression of these genes during the first days of exposure to CIS and TOP.

## 2. Results

### 2.1. Gene Expression Analysis in CIS- and TOP-Resistant Cell Lines

**Figure 1:**
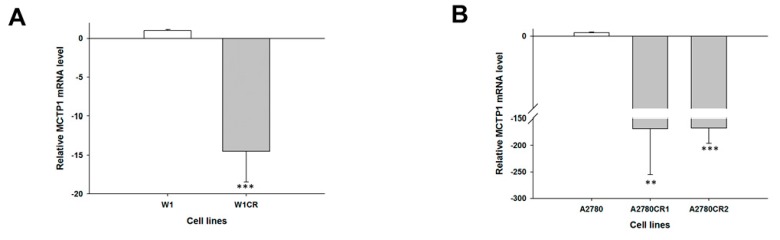

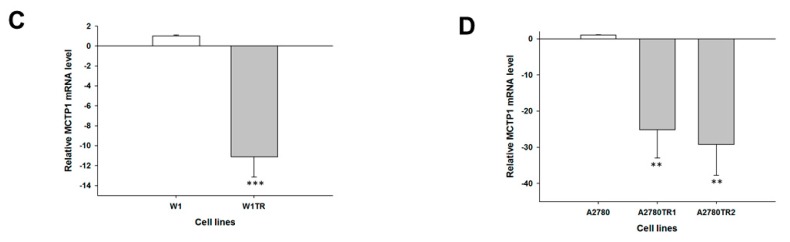
Expression analysis quantitative polymerase chain reaction (Q-PCR) of the *MCTP1* gene in: W1 (**A**); and A2780 (**B**) CIS-resistant cell sublines. Expression analysis of the *MCTP1* gene in: W1 (**C**); and A2780 (**D**) TOP-resistant sublines. The figure presents the relative gene expression in resistant cell lines (grey bars) with respect to the sensitive cell line (white bars), assigned as 1. The values were considered significant at ** *p <* 0.01 and *** *p* < 0.001.

**Figure 2:**
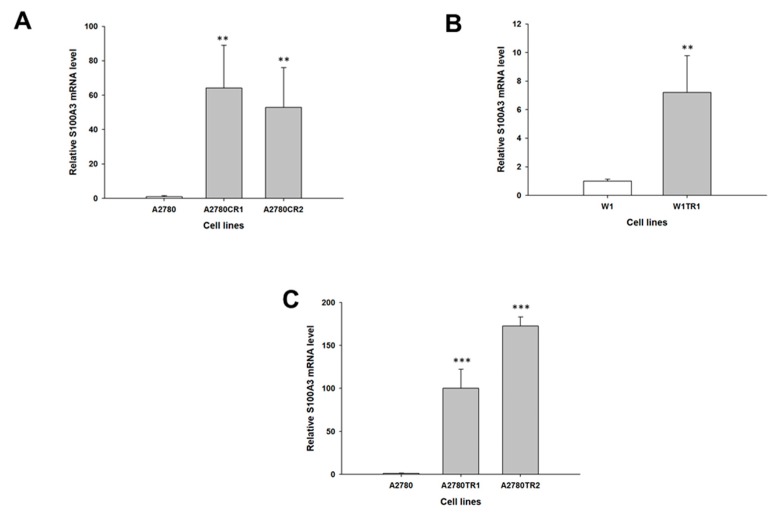
Expression analysis (Q-PCR) of the *S100A3* gene in: A2780 CIS-resistant cell lines (**A**); and W1 (**B**); and A2780 (**C**) TOP-resistant cell lines. The figure presents the relative gene expression in resistant cell lines (grey bars) with respect to the sensitive cell line (white bars), assigned as 1. The values were considered significant at ** *p <* 0.01 and *** *p* < 0.001.

**Figure 3:**
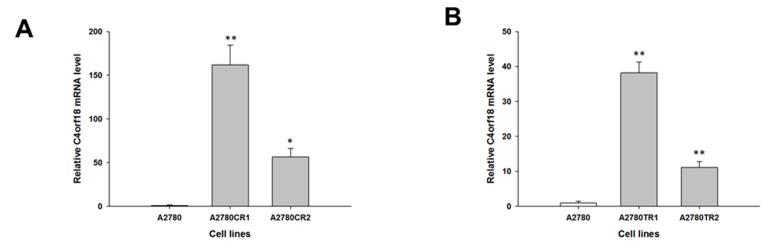
Expression analysis (Q-PCR) of the *C4orf18* gene in: A2780 CIS-resistant cell lines (**A**); and A2780 TOP-resistant cell lines (**B**). The figure presents relative the gene expression in resistant cell lines (grey bars) with respect to the sensitive cell line (white bars), assigned as 1. The values were considered significant at * *p <* 0.05 and ** *p* < 0.01.

**Figure 4:**
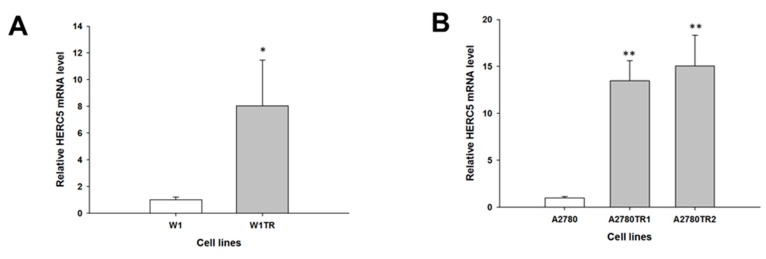
Expression analysis (Q-PCR) of the *HERC5* gene in: W1 (**A**); and A2780 TOP-resistant cell lines (**B**). The figure presents relative the gene expression in resistant cell lines (grey bars) with respect to the sensitive cell line (white bars), assigned as 1. The values were considered significant at * *p <* 0.05 and ** *p* < 0.01.

Our microarray data (not shown) suggest that the *MCTP1*, *S100A3*, *C4orf18* and *HERC5* genes may be involved in CIS and TOP resistance. The gene expression levels of *MCTP1*, *S100A3*, *C4orf18* and *HERC5* were examined to determine whether the CIS resistance and TOP resistance in our cell lines are associated with the changed expression of these genes. We observed a statistically significant decrease in *MCTP1* transcript levels in the W1CR cell line (*p* < 0.001) (Figure 1A) and in both A2780 CIS-resistant cell lines (*p* < 0.01 in the A2780CR1 cell line and *p* < 0.001 in the A2780CR2 cell line) (Figure 1B). However, in A2780 CIS-resistant cell lines, downregulation of the *MCTP1* transcript was much higher than in W1 CIS-resistant cell lines (approximately 150-fold vs. 15-fold). Decreased expression of *MCTP1* was also observed in cell lines resistant to TOP, both W1TR (*p* < 0.001) (Figure 1C) as well as in A2780TR1 and A2780TR2 (*p* < 0.01) (Figure 1D). Increased expression of *S100A3* was seen in both A2780 CIS-resistant cell lines (*p* < 0.01) (Figure 2A) but not in the W1CR cell line (not shown). However, *S100A3* transcript level was upregulated in the W1TR cell line (*p* < 0.01) (Figure 2B) as well as the A2780TR1 and A2780TR2 cell lines (*p* < 0.001) (Figure 2C). One should keep in mind that in A2780 TOP-resistant cell lines, upregulation of *S100A3* transcript levels was much higher than in the W1 TOP-resistant cell line (approximately 100- and 170-fold vs. seven-fold). A statistically significant increase in *C4orf18* transcript levels was observed in A2780 cell lines resistant to CIS (*p* < 0.01 in A2780CR1 cell line and *p* < 0.05 in A2780CR2 cell line) (Figure 3A). The *C4orf18* transcript level was also upregulated in both A2780 TOP-resistant cell lines (*p* < 0.01) (Figure 3B). Increased expression of the *HERC5* gene was characterized only for TOP-resistant cell lines. In the W1TR cell line, we observed an approximately eight-fold increase in *HERC5* mRNA levels (*p* < 0.01) (Figure 4A). Expression levels of *HERC5* mRNA increased more than 10 fold in both A2780 TOP-resistant cell lines (*p* < 0.01) (Figure 4B).

### 2.2. Early Response to CIS and TOP Treatment in Ovarian Cancer Cell Lines 

The second part of our study focused on the early response to CIS and TOP treatment. In these experiments, drug-sensitive cell lines W1 and A2780 were treated with low concentrations of CIS (250 ng/mL and 500 ng/mL) and TOP (10 ng/mL and 20 ng/mL) for 24, 48 and 72 h. Then, changes in gene expression were investigated.

**Figure 5:**
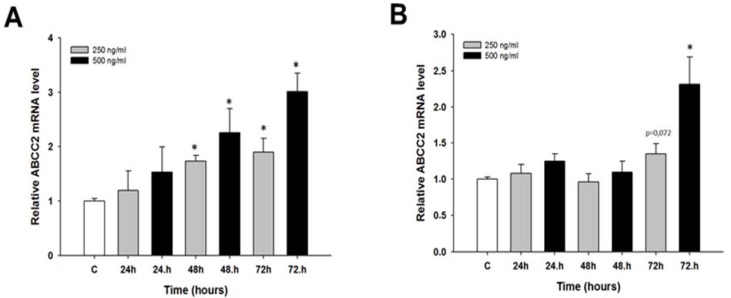
Expression analysis of the *ABCC2* gene in: W1 (**A**); and A2780 (**B**) ovarian cancer cell lines. The figure presents the relative gene expression in CIS-treated cells (grey and black bars) with respect to the untreated control (white bars), assigned as 1. The values were considered significant at * *p <* 0.05.

The expression levels of the *ABCC2* gene were investigated to determine if CIS can induce *ABCC2* expression in the first days of treatment. In the W1 cell line, we observed a concentration-dependent increase in the *ABCC2* transcript level after 48 and 72 h of treatment (*p* < 0.05) (Figure 5A). In contrast, in the A2780 cell line, a statistically significant increase in the *ABCC2* transcript level was observed only after 72 h of treatment at a CIS concentration of 500 ng/mL (*p* < 0.05) (Figure 5B).

**Figure 6:**
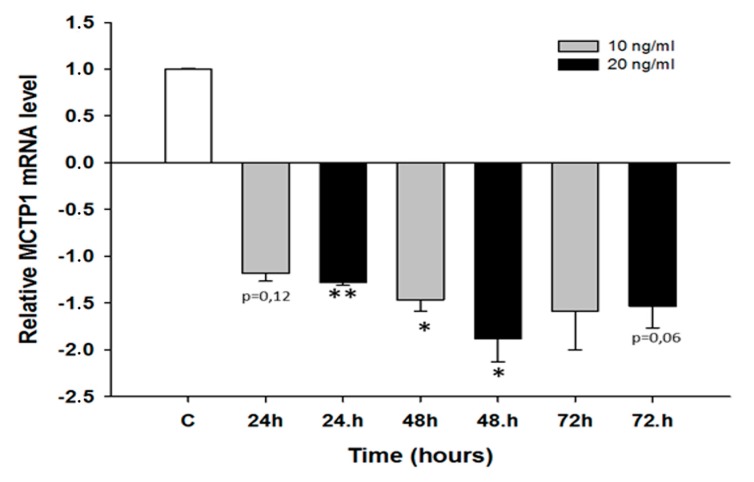
Expression analysis of the *MCTP1* gene in W1 ovarian cancer cell line. The figure presents the relative gene expression in TOP-treated cells (grey and black bars) with respect to the untreated control (white bars), assigned as 1. The values were considered significant at * *p <* 0.05 and ** *p* < 0.01.

**Figure 7:**
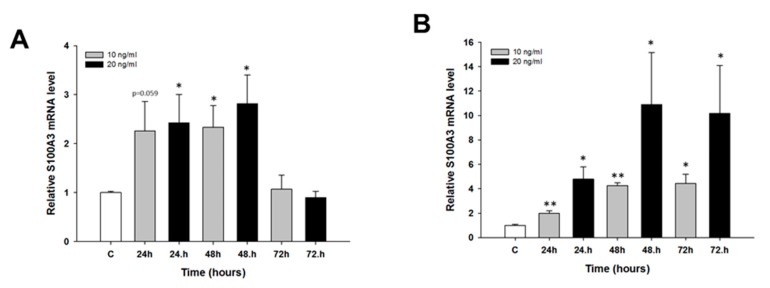
Expression analysis of the *S100A3* gene in: W1 (**A**); and A2780 (**B**) ovarian cancer cell lines. The figure presents the relative gene expression in TOP-treated cells (grey and black bars) with respect to the untreated control (white bars), assigned as 1. The values were considered significant at * *p <* 0.05 and ** *p* < 0.01.

**Figure 8:**
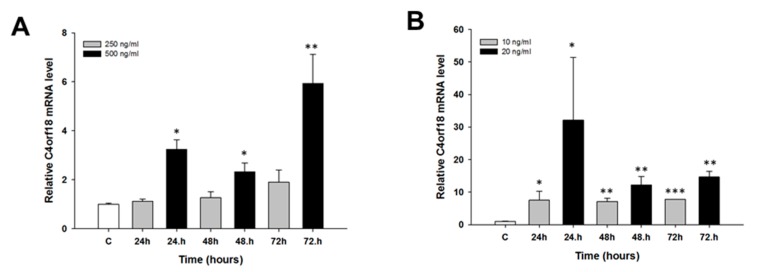
Expression analysis of the *C4orf18* gene in A2780 cell line. The figure presents the relative gene expression in: CIS-treated (**A**); and TOP-treated (**B**) cells (grey and black bars) with respect to the untreated control (white bars), assigned as 1. The values were considered significant at * *p <* 0.05, ** *p* < 0.01 and *** *p* < 0.001.

**Figure 9:**
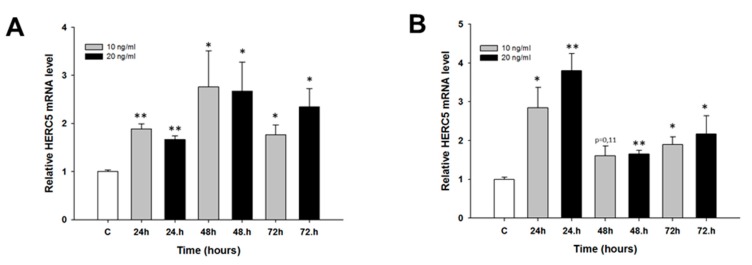
Expression analysis of the *HERC5* gene in: W1 (**A**); and A2780 (**B**) ovarian cancer cell lines. The figure presents the relative gene expression in TOP-treated cells (grey and black bars) with respect to the untreated control (white bars), assigned as 1. The values were considered significant at * *p <* 0.05 and ** *p* < 0.01.

We did not observe any changes in *MCTP1* mRNA levels after CIS treatment (not shown). TOP treatment resulted in the downregulation of *MCTP1* mRNA levels only in the W1 cell line. Although the *MCTP1* transcript level was decreased at all time points and at both TOP concentrations, a statistically significant decrease was observed only at 10 ng/mL after 24 h of treatment (*p* < 0.01) as well as at both 10 ng/mL and 20 ng/mL after 48 h of treatment (*p* < 0.05) (Figure 6). Similarly, changes in *S100A3* mRNA levels were observed only after TOP treatment. In the W1 cell line, we observed a statistically significant increase in *S100A3* transcript levels after 48 h of treatment at both TOP concentrations (*p* < 0.05) and after 24 h of treatment at a concentration of 20 ng/mL (*p* < 0.05). After 24 h of treatment with a concentration of 10 ng/mL, the increase was close to significant (*p* = 0.059). After 72 h, we did not observe significant changes in *S100A3* mRNA levels (Figure 7A). A greater increase in *S100A3* transcript levels was observed in the A2780 cell line. In this cell line, we observed a statistically significant increase (*p* < 0.05 or *p* < 0.01) at each time point and at both TOP concentrations. The increase seems to be dependent on the TOP concentration, and the maximum level was observed after 48 h and 72 h of treatment, with an approximate 10-fold increase (Figure 7B). Expression of the *C4orf18* gene increased in response to short time exposure, to both CIS and TOP, in the A2780 cell line. CIS treatment led to a statistically significant increase in the *C4orf18* transcript level after 24, 48 and 72 h of treatment but only at a concentration of 500 ng/mL (Figure 8A). A statistically significant increase in the *C4orf18* mRNA level was observed at both TOP concentrations and at each time point. We observed a 7–14-fold increase, with the exception of 24 h at concentration of 20 ng/mL, when a 32-fold increase was observed (Figure 8B). In all cases, a greater increase in mRNA levels was observed at a TOP concentration of 20 ng/mL. Overexpression of *HERC5* genes in the W1 cell line was observed after 24 h (*p* < 0.01) as well as after 48 and 72 h of treatment (*p* < 0.05) (Figure 9A). We also observed a statistically significant increase in *HERC5* mRNA levels in the A2780 cell line (with the exception of 10 ng/mL after 48 h). After 24 h of treatment, we observed higher expression levels than those after 48 and 72 h of treatment (Figure 9B).

## 3. Discussion

In this study, we investigated the expression of new genes that may be related to resistance towards CIS and TOP in ovarian cancer cell lines. CIS is the most important drug used in the first line of ovarian cancer chemotherapy [3], and TOP is used in the second line of ovarian cancer chemotherapy in case of platinum and taxane resistance in patients [5]. Previously, we described the expression of “old” genes related to CIS or TOP resistance in investigated cell lines [17,18,19,20]. Recently, we detected the expression of some “new” genes that may be related to drug resistance in ovarian cancer [19,25]. Here, we describe the expression of four new genes in established and primary responses to CIS and TOP treatment. In CIS- and TOP-resistant cell lines, derived from both W1 and A2780 cell lines, we observed decreased expression of the *MCTP1* gene. These results suggest that downregulation of this gene is specific to the resistance to both CIS and TOP in the investigated cell lines. MCTP1 is poorly described in the literature. MCTP1 belong to proteins containing a C2 Ca^2+^ binding motif, which is involved in membrane trafficking/exchange processes such as receptor trafficking, vesicle formation, and cell migration [26,27]. Downregulation of the expression of this gene suggests that these processes may be affected in the CIS and TOP resistant cell lines. Thus far, the expression of MCTP1 was investigated only in colorectal cancer with differential expression but without any correlation to clinical data [28]. Thus, the role of MCTP1 expression in cancer and cancer drug resistance requires further investigation. Because *MCTP1* gene was downregulated in all drug resistant cell lines, the restoration of its expression using overexpression system should restore drug sensitivity and better explain its role in drug resistance.

Another investigated gene was *S100A3*, which is involved in cell cycle progression and differentiation [29]. Increased expression of this gene was observed in both CIS- and TOP-resistant variants of the A2780 cell line and in the W1TR cell line. Increased expression in all TOP-resistant cell lines suggests that expression of the *S100A3* gene may be specific to resistance towards these agents. In the case of CIS, the role of *S100A3* may be cell line specific. *S100A3* expression has been reported in many cancers. In head and neck cancers, expression of the *S100A3* gene is upregulated in comparison to normal mucosa [40]. Immunohistochemical staining shows cell membrane and cytoplasmic expression of *S100A3* in human colorectal cancer and surrounding normal tissue; however, expression is much higher in tumor tissue [33]. A similar observation was made in human hepatocellular carcinoma (HCC) at both the transcript and protein levels. HepG2 cells also shows increased expression of *S100A3* in comparison to primary human hepatocytes [34]. A more prominent role of *S100A3* was observed in gastric and prostate cancers. Liu et al. showed that, in gastric cancer, the expression of *S100A3* was upregulated in comparison to that in adjacent non-tumor tissue. Furthermore, *S100A3* mRNA levels correlated with tumor differentiation, with low expression in well- and moderate-differentiated tumors and high expression in poorly differentiated ones. Expression was also higher in TNM stages III and IV than in stages I and II [32]. Kang et al. demonstrated that castration-resistant prostate cancer cell lines PC3 and DU145 present much higher expression levels of *S100A3* than do normal prostate cell line PNT2 and hormone-sensitive prostate cancer cell line LNCaP. Downregulation of *S100A3* expression using siRNA leads to apoptosis and reduced migratory and invasive properties of these cells and is associated with decreased expression of some metalloproteinases. In in vivo studies, tumor growth was significantly reduced in mice injected with PC3 and DU145 cells transfected with *S100A3* shRNA, in comparison to the control [41]. In similar way, downregulation of *S100A3* gene in drug resistant cell lines should better explain the significance of its increased expression in drug resistance phenomena. Increased expression of *S100A3* in different cancers suggests a role for it in cancer development. Higher expression in poorly differentiated and in metastatic tumors suggests that *S100A3* plays a role in drug resistance, since poorly differentiated tumors as well as metastatic ones are usually more resistant to chemotherapy than are well differentiated primary tumors.

Expression of the *C4orf18* gene in both A2780 CIS- and TOP-resistant cell lines suggests that *C4orf18* may play a specific role in drug resistance in A2780 drug-resistant sublines. Because of the lack of any other data in the literature, it is difficult to precisely define the role of *C4orf18* in drug resistance. However, the increased expression in cell lines resistant to drugs from the first and second lines of chemotherapy should be an invitation to other investigators to check its expression in cancers.

*HERC5* gene expression was upregulated in all TOP-resistant cell lines, suggesting a role for it in resistance towards this cytotoxic agent. *HERC5* is part of the ING15 protein degradation system, where *HERC5* plays the role of an E3 protein ligase that mediates the ISGylation of protein targets [35,36]. It is believed that the ISG15 system has low activity in normal tissue. In contrast, increased expression of proteins from this system was reported in prostate cancer tissue in comparison to control tissue [39]. Increased expression of the ISGN15 system has also been reported in pancreatic [42], breast [43], and bladder [44] tumors. Because it is known that expression of all proteins from this system is INF dependent [35], it could be suspected that *HERC5* should also be increased in these cancers. On the other hand, in NSCLC cancer, *HERC5* downregulation correlates with poor survival [45]. In contrast to our study, Desai et al. showed that higher expression of the ISG15 system increases sensitivity to camptothecin (CPT) a topotecan analogue, and downregulation of this system leads to increased resistance to camptothecin [46]. Thus, the expression of *HERC5* and other parts of the ISG15 protein degradation system seems to be tumor type specific. To further prove the role of *C4orf18* and *HERC5* genes in drug resistance, knock down experiments should be performed and the drug sensitivity/resistance should be checked.

In the second part of our research, we were interested in whether the same genes are expressed after first exposure to CIS and/or TOP treatment as those at the beginning of drug-resistance development. Two of the most important genes in resistance to CIS and TOP are *ABCC2* and *ABCG2*, encoding the MRP2 and BCRP proteins, respectively [47,48]. Previously, we reported *ABCC2* expression in CIS-resistant variants of A2780 [17,18] and W1 [20,49] cell lines. Here, we observed an increased expression of the *ABCC2* gene after 48 and 72 h of exposure in the W cell line and increased expression after 72 h of CIS treatment in the A2780 cell line. Increased expression after a short time of exposure confirms the significance of *ABCC2* gene in CIS resistance. Previously, we also observed increased expression of *ABCG2* gene, after a short time of exposure to TOP, in ovarian cancer cell lines [19]. Thus, the first response to the cytotoxic agent seems to indicate the direction of response to the drug. Following that, we were interested in whether genes described in drug-resistant cell lines can also be expressed after a short time of exposure to the investigated cytotoxic agents.

Expression of the *MCTP1* gene does not seem to play any role in the early response to CIS treatment, since we did not observe any changes in the expression of this gene after a short time of exposure. In contrast, expression of *MCTP1* was downregulated in the W1 cell line after TOP treatment, suggesting a role for *MCTP1* in the early response to TOP. Because *MCTP1* expression has not been described in the context of drug resistance so far, it is difficult to explain these results.

CIS treatment for a short time also did not induce any changes in the *S100A3* gene, suggesting that the *S100A3* gene is not important in the early response to this agent. In contrast, we observed increased expression of the *S100A3* gene after TOP treatment in both cell lines. Expression of *S100A3* seems to be important at the beginning of treatment in the W1 cell line. Dose- and time-dependent increases in the A2780 cell line suggest that *S100A3* expression plays an important role in TOP resistance in this cell line. Because *S100A3* was also increased in all TOP-resistant cell lines, it seems to be related to resistance towards this agent.

Similar to the established response, expression of the *C4orf18* gene in primary response to CIS and TOP treatment was observed only in the A2780 cell line. Increased expression was observed in response to higher concentrations of both CIS and TOP at each time point. Thus, expression of this poorly described gene seems to play a role in ovarian cancer drug resistance. To further explain the role of *C4orf18* in drug resistance, its expression should be confirmed in another ovarian cancer cell lines and in ovarian cancer tissue.

Another gene that seems to be related to TOP resistance is *HERC5.* Because *HERC5* expression increased in all TOP-resistant cell lines, we checked its early response to TOP. In both W1 as well as A2780 cell lines, we observed an increased expression of the *HERC5* gene at all time points. This suggests that *HERC5* may play an important role in resistance to this agent.

We are aware that our results have some limitations. Firstly, to better explain the significance of investigated genes in drug resistance the expression at protein level using Western blot should be performed. In our previous study [20,24] using our experimental system (drug sensitive—drug resistant cell lines), we had very high correlation between expression at mRNA and protein level, so we can suppose with high probability that this could also be true in case of these genes. However, the correlation between transcript and protein level is not always observed so in continuation of our research we will also make Western blot experiments. The second limitation of this study is that we did not perform knock down or overexpression experiments as we mentioned above. These kinds of experiments are very important for explaining the direct function of investigated genes and encoding proteins and will be made as a continuation of this research. In the future, we also plan to check the significance of investigated genes in drug resistance in in vivo experiments using animals. As an example of such study, Dong et al. investigated patient derived xenografts (PDX) of ovarian high-grade serous carcinoma (HGSC) in NOD and NSG mice [50]. In vivo study using cell lines with knock down or overexpression of investigated genes should better explain the significance of their expression in drug resistance. In contrast to standard cell culture condition where cells develop mainly cell specific mechanism of drug resistance in in vivo study cells form tumor tissue like structure. In tumor tissue, other mechanisms of drug resistance such as cell adhesion mediated drug resistance (CAM-DR) can be developed [51]. Thus, in vivo experiments should expand knowledge about the role of investigated genes in drug resistance.

The new proteins can be considered as a new target in ovarian cancer therapy. Because the increased expression of *S100A3*, *C4orf18* and *HERC5* genes was present in drug resistance cell lines it could be interested to check if new chemical agents or natural compounds can overcome this resistance. Recently, a new chemical agent NT1014 was used by Zang and coworkers in in vitro as well in vivo study using ovarian cancer cell lines. It inhibited cell proliferation, G1 cell cycle arrest and apoptosis in vivo and ovarian cancer grow in vitro [52]. Thus, it would be very interesting to check if new chemical agents can abrogate drug resistance in our cell lines.

Another therapeutic approach that can be effective in case of drug resistant cancers is immunotherapy. The chimeric antigen receptor T (CAR-T) cell therapy is a new strategy in adoptive antitumor treatment. CAR-T cells can eliminate tumor cells by interacting with the tumor-associated antigens. HER2, mesothelin and CA125 are a potential target to these lymphocytes in ovarian cancer [53]. Other strategy in immunotherapy are checkpoint blockade immunotherapies. In these therapies antibody against CTLA-r or PD-1 can restore and increase cytotoxic T cell response against chemotherapy resistant cancers [54].

In summary, we hope that, in the future, different strategies that increase effectiveness of chemotherapy will be used together and eventually cancer will become a treatable disease in most cases.

## 4. Materials and Methods

### 4.1. Reagents

CIS and TOP were obtained from Sigma (St. Louis, MO, USA). RPMI-1640 and MEM medium, foetal bovine serum, antibiotic-antimycotic solution, and l-glutamine were also purchased from Sigma (St. Louis, MO, USA).

### 4.2. Cell Lines and Cell Culture

In our study, we used two ovarian cancer cell lines: the established ovarian cancer cell line A2780 and the primary ovarian cancer cell line W1. The human ovarian carcinoma A2780 cell line was purchased from (ATCC, Manassas, VA, USA). A2780 sublines that were resistant to CIS [A2780CR1 and A2780CR2 (A2780 cisplatin resistant)] and TOP [A2780TR1 and A2780TR2 (A2780 topotecan resistant)] were generated by exposing A2780 cells to CIS or TOP at incrementally increasing concentrations. The human primary ovarian cancer cell line W1 was established using ovarian cancer tissue obtained from an untreated patient. The W1 sublines resistant to CIS [W1CR (W1 cisplatin resistant)] and TOP [W1TR (W1 topotecan resistant)] were obtained by exposing W1 cells to CIS or TOP at incrementally increasing concentrations. The final concentrations used for selecting the resistant cells were 1000 ng/mL of CIS and 24 ng/mL of TOP and were twofold higher than the plasma concentrations of CIS and TOP two hours after intravenous administration. The increase in resistance according to parental drug sensitive cell lines were as follows: 8.9-fold for W1CR vs. W1; 20.0-fold for W1TR vs. W1; 4.1-fold for A2780CR1 vs. A2780 and 3.3-fold for A2780CR2 vs. A2780; 59.6-fold for A2780TR1 vs. A2780 and 48.5-fold for A2780T2 vs. A2780 as described previously [18,20].

All the cell lines were maintained as monolayers in complete medium [MEM (A2780), and RPMI-1640 medium (W1) supplemented with 10% (*v/v*) foetal bovine serum, 2 pM l-glutamine, penicillin (100 units/mL), streptomycin (100 units/mL) and amphotericin B (25 μg/mL)] at 37 °C in a 5% CO_2_ atmosphere.

### 4.3. Incubation of Cells with CIS or TOP

In time course experiments, the W1 and A2780 cell lines were treated with CIS at a concentration of 250 ng/mL and 500 ng/mL or TOP at a concentration of 10 ng/mL and 20 ng/mL. The starting cell concentration was 0.5 × 10^6^ (W1 and A2780) in 1 mL of medium per well of a 6- well plate. Cell count and viability were determined before the cells were used in the different assays. Viability was determined by the trypan blue exclusion criteria. Cells were harvested and used for RNA isolation 24, 48 and 72 h after treatment.

### 4.4. Examination of Gene Expression by Q-PCR

Changes in *MCTP1*, *S100A3*, *C4orf18*, *HERC5* and *MRP2* gene expression in W1 and A2780 as well as in the CIS- and TOP-resistant cell lines were examined. RNA was isolated using a Gene Matrix Universal RNA purification kit (EURx Ltd., Gdańsk, Poland), as described by the manufacturer’s protocol. Reverse transcription was performed using the M-MLV reverse transcriptase (Invitrogen) as described in the manufacturer’s protocol using a thermal cycler (Veriti 96 well Thermal Cycler, Applied Biosystems, 850 Lincoln Centre Drive, Foster City, CA 94404, USA). Two micrograms of RNA were used for cDNA synthesis. Real-time PCR was performed using an Applied Biosystems PCR System (7900HT Fast Real-Time PCR System), Maxima SYBR Green/ROX qPCR Master Mix (Thermo Fisher Scientific, Waltham, MA, USA) and sequence-specific primers, as indicated in Table 1. Glyceraldehyde-3-phosphate dehydrogenase (*GADPH*), β-actin, hypoxanthine-guanine phosphoribosyltransferase 1 (*HRPT1*) and beta-2-microglobulin (*β2M*) served as the normalizing genes (geometric mean) against which changes in the expression of examined genes were compared. Gene expression was analyzed using the relative quantification (RQ) method. RQ estimates the differences in the level of gene expression against a calibrator (RQ of the calibrator = 1). The drug sensitive cell lines (W1 and A2780) were used as the calibrators. The analysis was conducted by employing the standard formula: RQ = [sample (drug-resistant line) calibrator (drug-sensitive line)]. The graphs were made using Sigma Plot 11.0 (Systat Software GmbH Schimmelbuschstrasse 25 D-40699, Erkrath, Germany).

For amplification, 12.5 μL of Maxima SYBR Green/ROX qPCR Master Mix (Fermentas), 1 μL of each primer (Oligo, Warsaw, Poland) (Table 1), 9.5 μL of water, and 1 μL of cDNA solution were mixed together. One RNA sample from each preparation was processed without the RT-reaction to act as a negative control in the subsequent PCR. Sample amplification included a hot start (95 °C, 15 min) followed by 45 cycles of denaturation at 95 °C for 15 s, annealing at 60 °C for 30 s, and extension at 72 °C for 30 s. After amplification, melting curve analysis was performed to analyze the melting temperature of the product. The amplification products were also resolved by 3% agarose gel electrophoresis and visualized by ethidium bromide staining.

### 4.5. Statistical Analysis

The statistical analysis was performed using Microsoft Excel software. The statistical significance of the differences was determined by applying Student’s *t*-test.

## 5. Conclusions

In summary, our results show the expression of new genes in response to CIS and TOP treatment in ovarian cancer cell lines. Decreased expression of the *MCTP1* gene in all resistant cell lines suggests a non-specific role for *MCTP1* in drug resistance. Expression of the *C4orf18* gene was characteristic for A2780 drug-resistant sublines. The significance of these genes in the resistance to cytotoxic agents was not described previously and should be further investigated. In contrast, increased expression of the *S100A3* and *HERC5* genes was described in different cancers. Our results suggest that these genes may also be associated with the development of drug resistance in cancer.

## Figures and Tables

**Table 1:** Oligonucleotide sequences used for RQ-PCR analysis.

Transcript	Sequence (5’-3’ Direction)		ENST Number	Product Size
MCTP1	F	AGAACCTCAACCCTGTGTGG	00000312216	123 bp
	R	AGGCTGAGCCCATAAAGTCA		
S100A3	F	GTGCACCTTCCAGGAATACG	00000368713	121 bp
	R	ACATTCCCGAAACTCAGTCG		
C4orf18	F	GAGTACCCAAGCCTGAATCG	00000393807	137 bp
	R	ATCTTCCTTGCGAGGTCTGA		
HERC5	F	CTTCCTGCATGTGGTTTCCT	00000264350	128 bp
	R	AAACAGTGCCAGTGGGAAAG		
ABCC2	F	AGAGTCAAAGCCAAGATGCC	00000370449	105 bp
	R	ACAGAGCCTTCATCAACCAG		
GAPDH	F	GAAGGTGAAGGTCGGAGTCA	00000229239	199 bp
	R	GACAAGCTTCCCGTTCTCAG		
β-actin	F	TCTGGCACCACACCTTCTAC	00000331789	169 bp
	R	GATAGCACAGCCTGGATAGC		
HPRT1	F	CTGAGGATTTGGAAAGGGTG	00000298556	156 bp
	R	AATCCAGCAGGTCAGCAAAG		
B2M	F	CGCTACTCTCTCTTTCTGGC	00000558401	133 bp
	R	ATGTCGGATGGATGAAACCC		
